# Supplementary material for: A Global Screen for Assembly State Changes of the Mitotic Proteome by SEC-SWATH-MS
Source: Cell Syst. 2020 Feb 26;10(2):133–155.e6. doi: 10.1016/j.cels.2020.01.001 (PMC7042714; doi:10.1016/j.cels.2020.01.001)

O43237 | DC1L2_HUMAN | DYNC1LI2 DNCLI2 LIC2
Monomer MW [kDa]: 54.099 Monomer expected elution fraction: 46

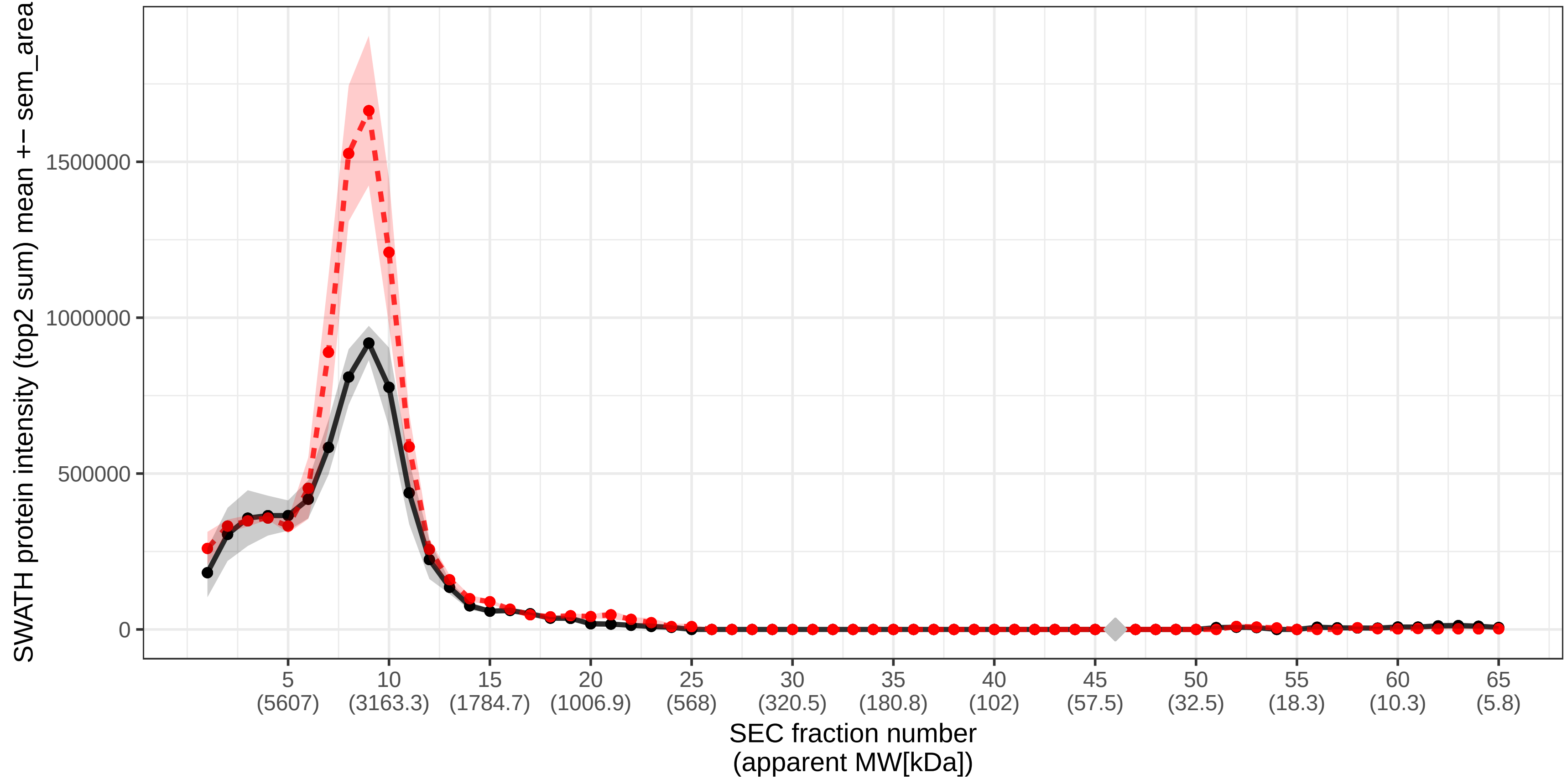

Supplement: Data S1. SEC-SWATH-MS Protein Chromatograms, Related to Figure 1 [file mmc6.zip › SECchrom_O43237_DC1L2_HUMAN_DYNC1LI2_DNCLI2_LIC2.pdf]
